# AI-based technology in Ophthalmology: the key to the future

**DOI:** 10.22336/rjo.2023.52

**Published:** 2023

**Authors:** Consuela-Mădălina Gheorghe

**Affiliations:** * Philologist, Certified translator

The advent of deep learning (DL), machine learning (ML), and artificial intelligence (AI) technologies in recent years has brought about a dramatic shift in the way of life in modern civilization. Artificial intelligence is a multifaceted technology that includes ML, DL, and sophisticated algorithms, among other components.

Up until the present, AI has been used in different fields of medicine such as Ophthalmology, Radiology, and Dermatology.

AI based on deep learning (DL) has acquired a great interest worldwide in recent years because DL has already been used in image recognition, speech recognition, natural language processing, and even in healthcare (Ting DSW, Pasquale LR, Peng L, Campbell JP, Lee AY, Raman R, Tan GSW, Schmetterer L, Keane PA, Wong TY. Artificial intelligence and deep learning in ophthalmology. Br J Ophthalmol. 2019 Feb; 103(2):167-175. doi: 10.1136/bjophthalmol-2018-313173. Epub 2018 Oct 25. PMID: 30361278; PMCID: PMC6362807).

The AI program known as ChatGPT, is a system developed by OpenAI and seems to be one of the latest advances made in AI, being known and used by more people in the world to aid in different fields and everyday life. This means that if we are looking for ophthalmological services online, we do not have to search for all such services on each website, the algorithm gathers all the information for us and we can easily decide regarding which service we choose. Thus, we spend less time searching and finding complex information online about a service we are interested in.

Artificial intelligence (AI) is already being utilized in ophthalmology to assist with data aggregation - gathering information from many sources and presenting it to the doctor to help with decision-making (https://www.healio.com/news/ophthalmology/20230404/ai-in-ophthalmology-from-code-to-clinic).

With its capacity to quickly quantify changes in values between ophthalmic imaging results and other test values, artificial intelligence (AI) has the potential to significantly advance precision medicine by enabling doctors to compare data between patient visits and make well-informed treatment decisions (https://www.healio.com/news/ophthalmology/20230404/ai-in-ophthalmology-from-code-to-clinic). Predictive analytics provide a huge prospect for glaucoma treatment decisions and monitoring of disease progression. AI might offer a fresh perspective to improve care in a personalized manner (https://www.healio.com/news/ophthalmology/20230404/ai-in-ophthalmology-from-code-to-clinic).

One area where AI has already greatly enhanced eye care is screening. The FDA approved Digital Diagnostics’ IDx-DR in 2018, making it the first AI tool for detecting diabetic retinopathy. The FDA subsequently approved the EyeArt system (Eyenuk) in 2020, making it the first device to test for cases of diabetic retinopathy that were potentially vision-threatening in addition to those that were only mild. 

Artificial intelligence (AI) technologies are used in ophthalmology to save thousands of eye photos and segment them into those that show a healthy eye and those that show a diseased eye using an algorithm. Additionally, the technology can determine whether a patient has an eye condition right away when a photo of their eye is entered (https://www.telefonica.com/en/communication-room/news/what-are-the-benefits-of-artificial-intelligence-in-ophthalmology/). 

Using the retinal light, these devices are also used to diagnose people at risk of cardiovascular disease. It is also used to diagnose macular degeneration and diabetic retinopathy. AI’s early detection can play a critical role in halting the spread of these diseases (https://www.telefonica.com/en/communication-room/news/what-are-the-benefits-of-artificial-intelligence-in-ophthalmology/).

Moreover, numerous AI-related studies have been conducted in the context of cataract surgery, since patients stand to gain from more accurate identification and individualized evaluations, perhaps saving their vision. The application of AI also ensures an early diagnosis of diseases and the customization of the treatments for each patient. 

AI also has some ethical aspects such as machine training, machine accuracy, patient-related, physician-related, shared, and roles of regulators. The solutions to these ethical issues seem to involve many factors (Abdullah YI, Schuman JS, Shabsigh R, Caplan A, Al-Aswad LA. Ethics of Artificial Intelligence in Medicine and Ophthalmology. Asia Pac J Ophthalmol (Phila). 2021 May-Jun 01;10(3):289-298. doi: 10.1097/APO.0000000000000397. PMID: 34383720; PMCID: PMC9167644). 

Furthermore, there are certain limitations to artificial intelligence in the screening of diabetic retinopathy, for example, resulting in the overdiagnosis and overtreatment of the patients. 

In conclusion, despite the benefits that AI brings to the healthcare field and the advances in technology, it will not take the place of experts very soon. Intelligent systems have been compared to human specialists in research, which is erroneous as technology complements physicians rather than replaces them. 


**Assist. Prof. Consuela-Mădălina Gheorghe, PhD, Philologist, Certified translator**


